# *Fusarium* phytopathogens as insect mutualists

**DOI:** 10.1371/journal.ppat.1011497

**Published:** 2023-07-27

**Authors:** Aileen Berasategui, Shounak Jagdale, Hassan Salem

**Affiliations:** 1 Mutualisms Research Group, Max Planck Institute for Biology, Tübingen, Germany; 2 Cluster of Excellence “Controlling Microbes to Fight Infections”, University of Tübingen, Tübingen, Germany; 3 Amsterdam Institute for Life and Environment, Vrije Universiteit, Amsterdam, the Netherlands; University of Maryland, Baltimore, UNITED STATES

## Abstract

As vectors of numerous plant pathogens, herbivorous insects play a key role in the epidemiology of plant disease. But how phytopathogens impact the metabolism, physiology, and fitness of their insect vectors is often unexplored within these tripartite interactions. Here, we examine the diverse symbioses forged between insects and members of the ascomycete fungal genus *Fusarium*. While *Fusarium* features numerous plant pathogens that are causal to diseases such as wilts and rots, many of these microbes also engage in stable mutualisms across several insect clades. Matching a diversity in symbiont localization and transmission routes, we highlight the various roles fusaria fulfill towards their insect hosts, from upgrading their nutritional physiology to providing defense against natural enemies. But as the insect partner is consistently herbivorous, we emphasize the convergent benefit *Fusarium* derives in exchange: propagation to a novel host plant. Collectively, we point to the synergy arising between a phytopathogen and its insect vector, and the consequences inflicted on their shared plant.

## 1. Introduction

**Fig 1:**
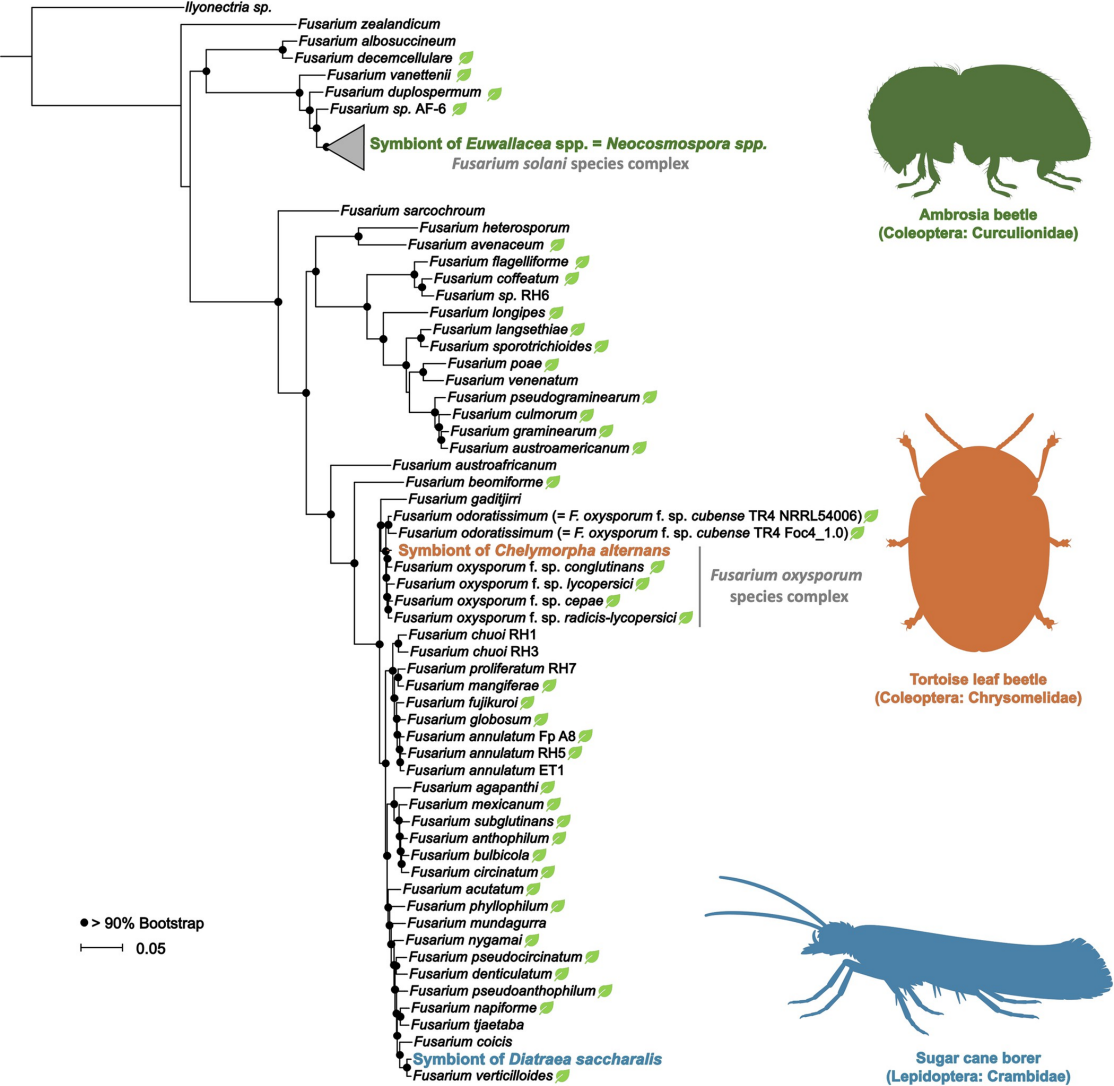
Independent origins of insect-fusarial symbioses. Maximum Likelihood phylogeny based on a concatenated alignment of 1,087 BUSCO genes (*Sordariomycete* lineage) revealing the phylogenetic placement of insect-associated fusaria relative to other *Fusarium* strains. Node coloration reflects bootstrap support >90%. Leaf drawings depict phytopathogenic strains. Insect schematics top to bottom: ambrosia beetle (green), tortoise leaf beetle (orange) and sugar cane borer (blue). Insect schematics created with BioRender.com and not to scale.

The genus *Fusarium* (*Sordariomycetes*: *Hypocreales*: *Nectriaceae*) represents one of the most ubiquitous plant-associated microbes [1]. Time-calibrated phylogenies indicate that fusaria originated approximately 91.3 Mya [2], coinciding with the radiation of flowering plants [3]. Members of the ascomycete genus are distributed worldwide and feature many plant pathogens impacting both managed and natural ecosystems [1,4,5]. By leveraging a remarkable arsenal of toxic secondary metabolites, fusaria cause several plant diseases, including wilts, blights, rots, and cankers [2]. But despite their known impact on plant health, members of the fungal genus are also increasingly recognized for their stable symbioses with insects (Fig 1), spanning a gradient of interactions outcomes. Here, we examine these associations and (1) highlight the repeated and independent origins of insect-fusarial symbioses, (2) outline the range of mutualistic benefits insects derive from these partnerships, and (3) emphasize the importance of the insect host in vectoring *Fusarium* to naïve plant populations, thereby expanding it transmission range.

### 1.1. *Fusarium* the cultivar

Fungal agriculture evolved independently across 3 orders of insects: once in ants, once in termites, and at least 7 times in beetles [6]. Ambrosia beetles (Curculionidae: Scolytinae and Platypodinae) represent a polyphyletic group of arthropods that construct galleries in the woody tissue of trees, where they inoculate and cultivate fungal gardens [6]. The beetles obligately depend on their fungal partners for nutrition, development, and reproduction [7,8]. The most advanced ambrosia farmers are beetles of the Xyleborini tribe, which impact woody plants of economic importance, such as avocado and fig trees [6,9]. Their symbiotic fusaria belong to the *Fusarium solani* species complex and represent a monophyletic clade that contains at least 12 lineages (Fig 1). The *Euwallacea-Fusarium* mutualism dates to the Oligocene-Miocene boundary (19 to 24 Mya), which overlaps with the adaptive radiation of the Xyleborini tribe (21 Mya) [10,11].

**Fig 2:**
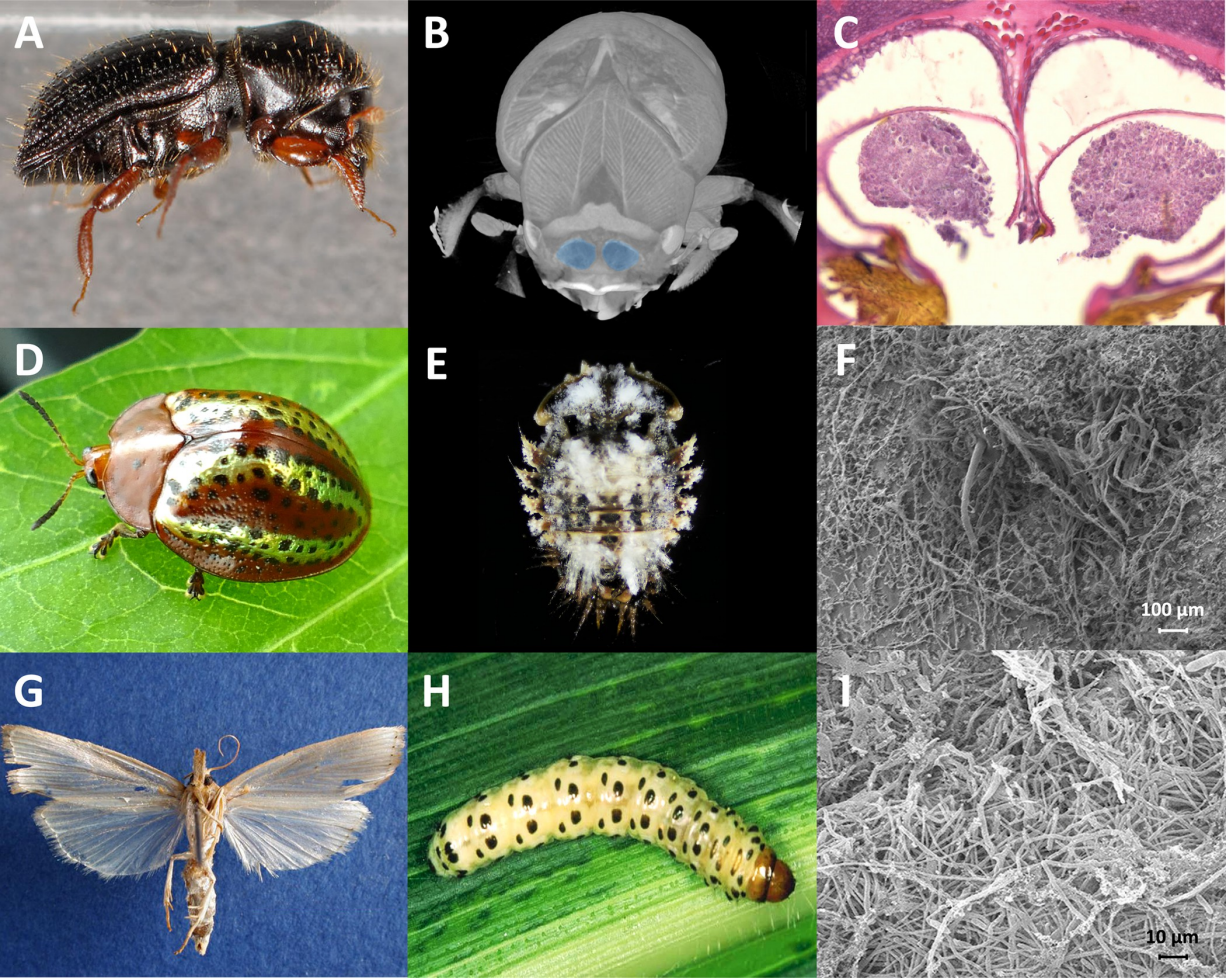
*Fusarium* localization in insects. (**A**-**C**) *Euwallacea validus* beetles rely for nutrition on fusarioid fungi that they harbor in pocket-like structures near the oral cavity called “mycangia” [12]. (**D**-**F**) *Chelymorpha alternans* beetles associate with *F*. *oxysporum*, which grows on the surface of their pupae during metamorphosis, protecting pupae from predation [22]. (**G**-**I**) *Diatraea saccharalis*, the sugarcane borer partners with *F*. *verticilloides*, which colonizes the caterpillar gut [37] and indirectly protects the larvae against parasitoid attack.

Ambrosia beetles acquire spores of their fungal partner from the natal garden and carry them in specialized pockets, or mycangia. These structures are critical for the propagation of a fungal inoculum in order to establish a garden in a new host plant [7]. In *Euwallacea*, these mycangia are located near the oral cavity (Fig 2A–2C) [12]. However, cophylogenetic studies demonstrate incongruent trees across the *Fusarium*-*Euwallacea* mutualisms, suggesting multiple host shifts during evolution [11]. Most cultivars have convergently evolved traits or structures that accumulate nutrients and increase their value for their insect farmers. This is the case of gongylidia (enlarged hyphal tips) in ants, nodules in termites, and swollen cells in ambrosia beetles associating with *Ophiostomatales* or *Microascales* [6,13,14]. In the same manner, ambrosial fusaria have evolved nutrient rich, club-shaped macroconidia that are consumed by the beetles [11]. Reflecting the ecological success of fungiculture, no reversals to nonsymbiotic lifestyles have been observed in ants, termites, or ambrosia beetles [13–15].

### 1.2. *Fusarium* as a defensive symbiont

Predators, pathogens, and other natural enemies represent strong selective pressures for organisms to evolve effective defensive strategies [16]. Symbioses with beneficial microbes serve as a rich source of defensive adaptations [17], allowing insects to safeguard immature stages such as eggs [18] and larvae [19], as well as valuable resources such as cultivars [20]. Similar to other herbivorous insects in the tropics, the risk of parasitism and predation can be high for the tortoise leaf beetle, *Chelymorpha alternans* (Chrysomelidae, Cassidinae) (Fig 2D) [21]. Larvae counter these threats by deploying fecal shields to fend off ants and parasitoid wasps [21]. Pupae, however, lack that adaptation and instead rely on microbial protection during an immobile developmental stage [22]. In addition to its nutritional endosymbionts [23–25], *C*. *alternans* hosts a stable mycobiome predominantly composed of *Fusarium oxysporum* (Fig 1) [22]. While *F*. *oxysporum* is present throughout the life cycle of the insect and is faithfully transmitted, the symbiont rapidly and conspicuously proliferates at the onset of pupation where it coats the host during metamorphosis (Fig 2E and 2F). Symbiont elimination results in increased predation under natural field conditions relative to symbiont-bearing pupae, demonstrating the protective nature of the association [22]. Chromosome-scale genome sequencing and assembly revealed that the symbiont possesses a haploid genome that is relatively reduced in size (48.6 Mb) compared to close relatives within the *F*. *oxysporum* species complex [22]. Despite signatures of reductive genome evolution common among many symbionts, *F*. *oxysporum* retained the metabolic capacity to produce at least 42 secondary metabolites. Many of these compounds, such as beauvericin and bikaverin, are known for their insecticidal properties and likely underpin the defensive partnership with tortoise beetles [22].

### 1.3. Indirect benefits in moths

Similar to beetles, lepidopteran insects frequently harbor phytopathogenic fusaria (Figs 1 and 2G–2I). Caterpillars of the stalk borer (*Eldana saccharina*) and the European corn borer (*Ostrinia nubilalis*), for instance, partner with a diversity of *Fusarium* species, including *F*. *verticilloides* [26,27], which are responsible for a number of rot diseases. Sugarcane borer caterpillars (*Diatraea saccharalis)* are also demonstrated to host *F*. *verticilloides* in what was initially thought to be an opportunistic interaction for the fungus. It was assumed that *F*. *verticilloides* exploits openings caused by the borer’s larvae to penetrate the plant stem and initiate infection, causing red rot disease [28]. However, the phytopathogen also colonizes the caterpillar’s digestive system, inducing morphological changes in the intestinal wall such as increasing its thickness and the length of microvilli [29]. In response to herbivore damage, sugarcane plants emit volatiles that attract *D*. *saccharalis* enemies, such as *Cotesia flavipes* parasitoids. Strikingly, *Fusarium*-infected plants attract fewer parasitoids than noninfected sugarcanes [30], demonstrating an indirect benefit to *D*. *saccharalis* for vectoring the fungus [30].

### 1.4. Herbivorous insects are vehicles for phytopathogen transfer

Due to their ecological diversity and their herbivorous habits, insects are important vectors of many plant diseases [31]. Vector-borne pathogens are predicted to (i) increase their dissemination by manipulating insect behavior and (ii) evolve to be less virulent to its vector over evolutionary time [31]. While the benefits insects derive from their fusarial partners are diverse, spanning nutrition to defense, the benefit of associating with an insect for the microbial partner is conserved: propagation to naïve plants, thereby expanding their ecological range.

In the process of vertically transmitting their fusarial cultivars to establish new gardens, ambrosia beetles disseminate their mutualistic partners to a diversity of host plants such as avocado, hazelnut, and fig trees, where they are responsible for wilt and dieback disease [9]. While *Fusarium* cultivars can survive on trees for up to 2 years, they cannot spread beyond the galleries, highlighting their strict reliance on ambrosia beetles for dispersal via specialized mycangia [32,33].

Following metamorphosis, tortoise beetles shed their *Fusarium*-rich pupal skin with their tarsi [21]. This behavior favors the contamination of beetle appendages with fungal hyphae and spores that are subsequently propagated to naïve plants when beetles transition to new feeding sites [22]. Once on the vascular tissues of a new host plant, the phytopathogen initiates infection, causing yellow wilt disease in sweet potato plants [22]. Additionally, the fungus could manipulate beetle behavior to promote its own dissemination, as demonstrated in mealworm beetles (*Tenebrio molitor*), which prefers to consume wheat grains infected with *F*. *proliferatum* [34] and continues to excrete spores in their feces weeks after their ingestion.

Revealing a more intimate tripartite interaction than previously envisioned, sugarcane plants up-regulate the production of defensive proteins upon *D*. *saccharalis* attack that do not negatively affect the insect but leads to fungal apoptosis [35,36]. Despite the deployment of antifungal plant defenses induced by the herbivore, *F*. *verticilloides* overall benefits from its interaction with the sugarcane borer by manipulating both its plant host and the insect to enhance its own transmission [37]. On the one hand, *F*. *verticilloides* emits volatile compounds that are attractive for the insect. But on the other hand, the fungus alters female oviposition preference. While females that are not colonized by *Fusarium* prefer to lay eggs on fungus-infected plants, females carrying the phytopathogen prefer to lay eggs on fungus-free plants. In both cases, the fungus increases its own dissemination [37].

### 1.5. Concluding remarks

Herbivorous insects are increasingly recognized for their role in the epidemiology of numerous plant pathogens, including diverse *Fusarium* species. While the physiological consequences of pathogen infection are well-characterized for the host plant, how these microbes affect the behavior, development, and overall fitness of their insect vectors remains underexplored in most cases. Given their tractability, insect–*Fusarium* interactions serve as emerging experimental model systems to improve our understanding of the ecology and evolution of plant–pathogen–vector associations.
